# A brief report on early sleep studies

**DOI:** 10.5935/1984-0063.20190144

**Published:** 2020

**Authors:** Monica Levy Andersen

**Affiliations:** Departamento de Psicobiologia, Universidade Federal de São Paulo Rua Napoleão de Barros, 925, 04024- 002, São Paulo SP, Brazil

A number of pioneering experimental sleep studies were performed in the 19^th^ century.^1^ In 1894, the Russian physician Marie de Manaceine submitted dogs to continuous stimulation to evaluate the effects of sleep deprivation (SD).^2^ The results revealed a fascinating discovery - sleep absence caused the puppies’ deaths. Four years later, Lambert Daddi and Giulio Tarozzi kept dogs awake by walking them until they died, which occurred after 9 to 17 days of SD.^3^ Interestingly, both studies revealed that SD provoked alterations in body temperature and blood cells combined with fatigue and small hemorrhages in the brain. The method used to promote SD by the authors was questioned by scientists because it involved physical activity (intense walking and handling) that has other biological repercussions.^1^ Stimulated by the previous findings, but concerned about the impact of physical activity, Cesare Agostini kept 2 dogs awake in metal cages to avoid physical fatigue and found that after 12 and 17 days they died, and their brains demonstrated degenerative changes.^4^ These results showed experimentally for the first time that sleep was essential to life. In addition to the sleep studies, Cesare Agostini also reported the cases of 2 people, whose behaviors became unusual and strange following SD - displaying extreme excitation, hallucinations, severe attention deficit, and inadequate emotional attitudes.^4^ All the symptoms disappeared after a period of sleep. These preliminary SD findings highlighted that sleep is an important and inherent physiological state required for survival.

The experiments to understand the effects of SD carried out on animals had some limitations, such as the possible confounding effects of stress, intense motor activity, isolation and the difficulty of establishing a matching control group with the same environmental conditions.

The evolutionary persistence of sleep, in the face of the apparent costs of increased vulnerability to predation and a reduction in time for foraging and other activities, suggests that it has an essential function.^5^ Although different animals have different rebound periods after SD, evolutionarily distant species share common sleep functions, including roles in development,^6^ metabolism^7^ and memory consolidation.^8^ One of the most recent theories is that sleep is associated with neural changes including consolidation of synaptic strength and the development of the central nervous system.^6^ Sleep behavior has been studied in a wide variety of animals with a central nervous system, including vertebrates, arthropods, ^9-11^ mollusks, ^12,13^ roundworms^14^ and flatworms.^15^ Studies of amphibians and fish are less common, although they could be helpful in clarifying the evolution of sleep states in vertebrates.^16,17^ Mammals are the most intensively studied group and, therefore, their mechanisms of sleep are better understood, although the daily amount of sleep required varies greatly among them^18^, with some species being able to maintain their waking performance and health with remarkably little sleep.^19^ Over recent decades, molecular and in vitro studies have also provided significant insights into sleep function and regulation.^5^

It is well known that sleep loss has considerable effects on biochemical and neurochemical processes, the immune system, body metabolism, physiological measures including electroencephalogram (EEG), energy metabolism, and thermoregulation.^1^ Although sleep has been intensively studied, and its mechanisms are better understood, it still seems to be underappreciated by humans, who have, in general, developed lifestyles that either by choice or necessity put pressure on the ability of individuals to get adequate sleep, which in turn can have serious implications for health

Sleep is a state that continues to challenge and intrigue researchers, even more than 100 years after the earliest animal sleep experiments. In this period, the study of both simple and complex organisms has contributed to a better understanding of the importance of sleep for health, and were key to the realization that sleep is a complex behavior that exercises a central role in the life of animals.

## References

[1] Bentivoglio M, Grassi-Zucconi G (1997). The History of Sleep Advances: The Pioneering Experimental Studies on Sleep Deprivation. Sleep.

[2] De Manaceine M (1894). Quelques observations expérimentales sur I'influence de I'insomnie absolue. Arch Ital Biol.

[3] Daddi L, Tarozzi G (1898). Sulle alterazioni degli elementi del sistema neroso centrale nell'insonnia sperimentale. Rivista di Patologia Nervosa e Mentale.

[4] Agostini C (1898). Sui disturbi psichici e sulle alterazioni del sistema nervoso centrale per insonnia assoluta. Rivista Sperimentale di Freniatria.

[5] Anafi RC, Kayser MS, Raizen DM (2019). Exploring phylogeny to find the function of sleep. Nat Rev Neurosci.

[6] Kayser MS, Biron D (2016). Sleep and development in genetically tractable model organisms. Genetics.

[7] Yurgel ME, Masek P, DiAngelo J, Keene AC (2015). Genetic dissection of sleep-metabolism interactions in the fruit fly. J Comp Physiol A.

[8] Seugnet L, Galvin JE, Suzuki Y, Gottschalk L, Shaw PJ (2009). Persistent short-term memory defects following sleep deprivation in a Drosophila model of Parkinson disease. Sleep.

[9] Kaiser W, Steiner-Kaiser J (1983). Neuronal correlates of sleep, wakefulness and arousal in a diurnal insect. Nature.

[10] Tobler I, Stalder J (1988). Rest in the scorpion - a sleep-like state?. Journal of Comparative Physiology A.

[11] Ramón F, Mendoza-Angeles K, Hernández-Falcón J (2012). Sleep in invertebrates: crayfish. Frontiers in Bioscience.

[12] Vorster APA, Krishnan HC, Cirelli C, Lyons LC (2014). Characterization of sleep in Aplysia californica. Sleep.

[13] Iglesias TL, Boal JG, Frank MG, Zeil J, Hanlon RT (2019). Cyclic nature of the REM sleep-like state in the cuttlefish Sepia officinalis. J Exp Biol.

[14] Nichols ALA, Eichler T, Latham R, Zimmer M (2017). A global brain state underlies C. elegans sleep behavior. Science.

[15] Omond S, Ly LMT, Beaton R, Storm JJ, Hale MW, Lesku JA (2017). Inactivity is nycthemeral, endogenously generated, homeostatically regulated, and melatonin modulated in a free-living platyhelminth flatworm. Sleep.

[16] Libourel PA, Herrel A (2016). Sleep in amphibians and reptiles: a review and a preliminary analysis of evolutionary patterns. Biological Reviews.

[17] Kelly ML, Collin SP, Hemmi JM, Lesku JA (2019). Evidence for sleep in sharks and rays: behavioural, physiological, and evolutionary considerations. Brain Behav Evol.

[18] Lesku JA, Roth TCII, Rattenborg NC, Amlaner CJ, Lima SL (2008). Phylogenetics and the correlates of mammalian sleep: a reappraisal. Sleep Medicine Reviews.

[19] Ungurean G, van der Meij J, Rattenborg NC, Lesku JA (2020). Evolution and plasticity of sleep. Current Opinion in Physiol.

